# Recursive partitioning analysis for survival stratification and early imaging prediction of molecular biomarker in glioma patients

**DOI:** 10.1186/s12885-024-12542-w

**Published:** 2024-07-09

**Authors:** Xian Xie, Chen Luo, Shuai Wu, Wanyu Qiao, Wei Deng, Lei Jin, Junfeng Lu, Linghao Bu, Hugues Duffau, Jie Zhang, Ye Yao

**Affiliations:** 1 Department of Biostatistics, School of Public Health & National Clinical Research Center for Aging and Medicine, Huashan Hospital, Fudan University, Shanghai, 200032 China; 2 Department of Neurosurgery, Huashan Hospital, Shanghai Medical College, Fudan University, Shanghai, 200040 China; 3 National Center for Neurological Disorders, Shanghai, 200052 China; 4 Shanghai Key Laboratory of Brain Function and Restoration and Neural Regeneration, Shanghai, 200040 China; 5 Neurosurgical Institute of Fudan University, Shanghai, 200052 China; 6 Department of Neurosurgery, The First Affiliated Hospital, Zhejiang University School of Medicine, Hangzhou, Zhejiang China; 7 Department of Neurosurgery, Gui de Chauliac Hospital, Montpellier University Medical Center, 80 Avenue Agustin Fliche, Montpellier, 34295 France; 8 Key Laboratory of Public Health Safety of Ministry of Education, Fudan University, Shanghai, 200032 China

**Keywords:** Glioma, Isocitrate dehydrogenase, Telomerase reverse transcriptase, VASARI, Recursive partitioning analysis

## Abstract

**Background:**

Glioma is the most common primary brain tumor with high mortality and disability rates. Recent studies have highlighted the significant prognostic consequences of subtyping molecular pathological markers using tumor samples, such as IDH, 1p/19q, and TERT. However, the relative importance of individual markers or marker combinations in affecting patient survival remains unclear. Moreover, the high cost and reliance on postoperative tumor samples hinder the widespread use of these molecular markers in clinical practice, particularly during the preoperative period. We aim to identify the most prominent molecular biomarker combination that affects patient survival and develop a preoperative MRI-based predictive model and clinical scoring system for this combination.

**Methods:**

A cohort dataset of 2,879 patients was compiled for survival risk stratification. In a subset of 238 patients, recursive partitioning analysis (RPA) was applied to create a survival subgroup framework based on molecular markers. We then collected MRI data and applied Visually Accessible Rembrandt Images (VASARI) features to construct predictive models and clinical scoring systems.

**Results:**

The RPA delineated four survival groups primarily defined by the status of IDH and TERT mutations. Predictive models incorporating VASARI features and clinical data achieved AUC values of 0.85 for IDH and 0.82 for TERT mutations. Nomogram-based scoring systems were also formulated to facilitate clinical application.

**Conclusions:**

The combination of IDH-TERT mutation status alone can identify the most distinct survival differences in glioma patients. The predictive model based on preoperative MRI features, supported by clinical assessments, offers a reliable method for early molecular mutation prediction and constitutes a valuable scoring tool for clinicians in guiding treatment strategies.

**Supplementary Information:**

The online version contains supplementary material available at 10.1186/s12885-024-12542-w.

## Background

Gliomas are the most common type of primary brain tumor, often characterized by aggressive infiltration into the surrounding brain tissue [1]. Despite multiple therapeutic interventions, including surgery, radiotherapy, and chemotherapy, the prognosis for patients afflicted with gliomas remains bleak [2, 3]. Such fatal outcomes inflict severe physical, psychological, and financial burdens on patients and their families [4, 5]. Many factors may affect the prognosis of glioma patients, including the patient’s age, functional status, and especially, the various molecular biomarkers [6, 7]. Recent advancements in molecular biology have revolutionized our understanding of gliomas, leading to the identification of key biomarkers that play a critical role in the pathophysiology of the disease. The fifth edition of the World Health Organization (WHO) Classification of Tumors of the Central Nervous System (WHO CNS5) has further emphasized the importance of these molecular signatures, integrating them into the diagnostic criteria and thereby influencing treatment paradigms [8–10].

In the clinical setting, comprehensive assessment of multiple molecular markers such as isocitrate dehydrogenase (IDH), 1p/19q, telomerase reverse transcriptase (TERT) promoter and O6-methylguanine-DNA methyltransferase (MGMT) promoter in patients is critical for clinicians when making comprehensive and accurate judgments about patients’ conditions. Of key importance is to consider the direction and magnitude of impact that different biomarkers have on survival, as well as their potential interactions [7]. Moreover, complex interactions between multiple biomarkers may work in synergy. For instance, IDH mutant (IDHmut) gliomas with 1p/19q codeletion often signal a better prognosis [11, 12], and the co-occurrence of IDH and TERT promoter mutations in oligodendrogliomas correlates with an improved survival rate, unlike the prognosis for tumors with TERT mutations (TERTmut) but without IDH mutations [10–12].

Traditional survival analysis methods, such as COX regression, may not adequately account for the interplay of multiple biomarkers [13], researches in recent years has proposed a variety of ensemble learning methods, such as soft Bayesian additive regression trees, for determining survival prediction when complex unknown effects are present under multiple covariates [14–17]. The recursive partitioning analysis (RPA) [18, 19] used in this study allows for the consideration of interactions and avoids over-classification while ensuring the effectiveness of the grouping, presenting the optimal grouping results in the form of a tree. This method has been effectively applied to various cancers [20, 21], including gliomas [20–22], allowing physicians to visualize and quickly comprehend the survival grouping profile, enabling them to make overall survival judgments about glioma patients accordingly.

The preoperative identification of molecular survival subgroups can enrich discussions with patients regarding their treatment plans, particularly concerning their postoperative quality of life and functional outcomes. However, the challenge lies in the fact that current methods for determining molecular status are invasive and time-consuming. Using imaging techniques may help to obtain molecular marker status preoperatively. Visually Accessible Rembrandt Images (VASARI) is a feature set composed of magnetic resonance imaging (MRI) features of glioma patients. These imaging features are easily observable and clinically interpretable [23, 24].

The objective of this study was to enable preoperative survival prediction, encourage tailored treatment, and further enhance patient choice of prognosis. In clinical practice, the goal of maximal safe resection is unquestionable with the aim of maximizing survival, although having been shown that patients with various molecular marker statuses differ in their surgical benefits [25, 26]. However, the acceptability of even short-term functional deficits may vary among patients with different expected survival times and job nature. By assessing patients’ molecular marker status and prognosis preoperatively, it may be possible to increase the involvement of patients and their families in the development of a surgical strategy for functional preservation, thereby helping to enhance the patient’s sense of control over their postoperative life. This refined and individualized development of surgical strategies can help to achieve a more ideal prognostic status and improve patients’ postoperative willingness to survive and quality of life while respecting their individual wishes.

To achieve preoperative survival risk grouping and guide treatment strategy, we sequentially collected cohort datasets and imaging datasets for survival risk grouping and molecular marker prediction, respectively. Employing RPA, we identified various risk groups based on molecular indicators including IDH, 1p/19q, TERT, and MGMT. We then used logistic regression to determine significant imaging features that correlate with these indicators, creating nomograms for predictive scoring. This combination of predictive scoring and survival trees equips clinicians with a powerful tool for preoperative risk assessment, leading to more personalized treatment strategies for glioma patients.

## Methods

### Cohort database

**Fig. 1:**
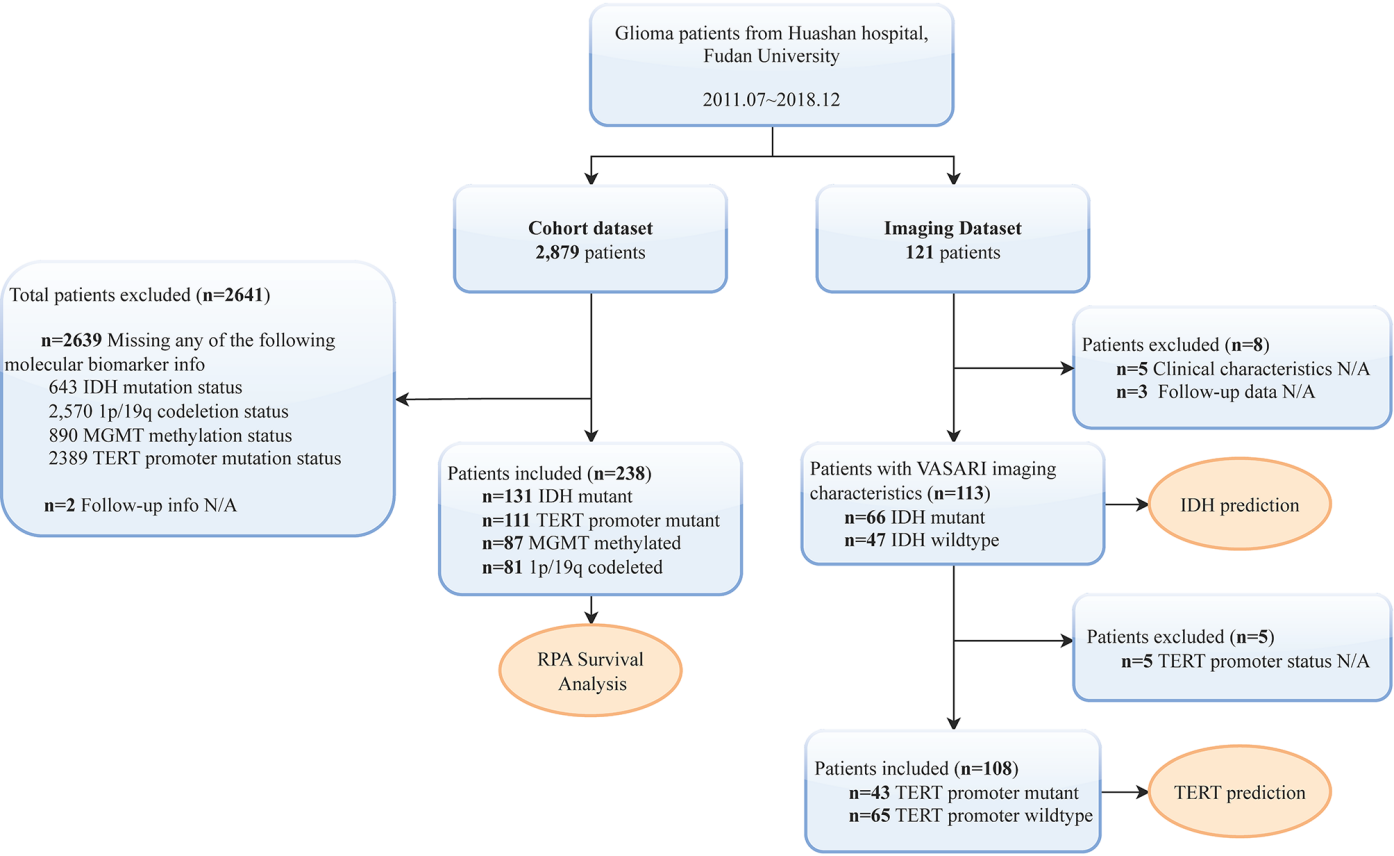
Flowchart. The relevant data were collected from the Department of Neurologic Surgery of Huashan Hospital, Fudan University, China. After excluding subjects with missing information, we obtained a cohort dataset for Recursive Partitioning Analysis (RPA) and an imaging dataset for molecular prediction, respectively. This work is split into two sections: first, we used the cohort dataset’s data to perform RPA, which allowed us to develop a survival grouping framework made up of molecular markers. Next, we performed imaging predictions of the molecular markers that were part of the survival grouping framework in the imaging dataset

A cohort of 2879 infiltrating glioma cases who underwent tumor resection was obtained from the Department of Neurologic Surgery, Huashan Hospital, Fudan University, between 2013.06 and 2018.12. Eligible subjects are required to have undergone molecular marker tests and have complete information on the molecular markers, including IDH, 1p/19q, TERT, and MGMT. Individuals without information on any of the molecular markers were considered ineligible and excluded. A total of 2639 participants with unavailable information on one or more molecular markers were excluded, as were 2 subjects with missing follow-up data, resulting in data from 238 subjects (117 WHO grade 2, 66 WHO grade 3, and 55 WHO grade 4) to classify the survival risk group (Fig. 1). We utilized Recursive Partitioning Analysis (RPA) employing the partitioning Deletion/Substitution/Addition (partDSA) algorithm with the aim of selecting and determining the multivariate combinations that most efficiently discriminate patient prognosis from all potential prognostic variables. Potential variables used to establish survival groups included gender, age, Karnofsky Performance Status (KPS) score, IDH mutation status, 1p/19q codeletion status, TERT promoter mutation status, and MGMT methylation status. Of these, age and KPS score were continuous variables, whereas the remaining five variables were all dichotomous, as follows: gender (male/female), IDH status (mutant/wild-type), 1p/19q status (codeletion/intact), TERT status (mutant/wild-type), and MGMT status (methylated/unmethylated). Less essential variables will be excluded during this procedure, leaving just the combination of variables that best differentiate patient survival to establish the survival risk group. The findings were visualized as a tree structure, allowing for the classification of subjects into various groups based on their risk of survival. The tree with the minimized five-fold cross-validated integrated Brier error was chosen. The leaves of the resultant tree defined the final risk groupings and the corresponding Kaplan-Meier curves were generated accordingly. Lower-grade gliomas are defined as grade 2–3 gliomas [27]. Overall survival (OS) was defined as the period from operation to patient death. The institutional review boards of Huashan Hospital, Fudan University, Shanghai, China, have provided ethical approval for this study (approval numbers KY2015-256 & KY2023-539).

### Imaging database

Age, gender, KPS score, and four molecular markers (IDH, 1p/19q, TERT, and MGMT) were among the seven variables that were added to the partDSA algorithm in the previous section. The algorithm then continuously screened the survival risk framework consisting of one or more of these seven variables, and the survival risk framework consisting of two variables, IDH and TERT, was finally selected by the algorithm as the optimal survival risk tree to classify glioma patients into the four groups. Based on the results of the preceding step of establishing survival risk groups, imaging predictions of the molecular markers involved in constituting the survival risk groups were performed. In the initial stage, RPA developed a survival risk tree utilizing a couple of molecular markers, IDH and TERT. Subsequently, we focused on leveraging imaging data to forecast the status of IDH and TERT mutations. Hence, clinical and imaging data were collected for 121 patients whose preoperative MRI (T1-weighted, Flair) images were available at the Department of Neurologic Surgery, Huashan Hospital, Fudan University from 2011.07 to 2018.04. Five of the 121 participants were excluded due to a lack of clinical information, and three were excluded due to a lack of follow-up information, leaving 113 subjects (71 WHO grade 2 and 42 WHO grade 3) in the IDH prediction. Five cases were excluded because no TERT promoter mutation information was provided, leaving 108 (67 WHO grade 2 and 41 WHO grade 3) for whom clinical and imaging data were used to predict TERT promoter mutations (Fig. 1).

We included clinical information on both chief complaints and onset symptoms. Glioma patients frequently complained of sudden disturbance or loss of consciousness, headache, dizziness, limb numbness, and so on. Epilepsy, headache, speech impairment, and visual impairment were among the first symptoms to appear. VASARI (https://wiki.nci.nih.gov/display/CIP/VASARI ) is a controlled vocabulary for characterizing the baseline visual features of human glioma images (features were described in detail in the supplementary **eMethods**). Based on preoperative MRI scans (T1-weighted, Flair), imaging phenotypes were identified and assessed by experienced neurosurgeons (J.Z. & L.B.) using the VASARI feature set. Following the two neurosurgeons’ comprehensive review of VASARI feature definitions and their subsequent agreement on standardizing assessment criteria, two physicians who were blinded to the patients’ clinical data independently conducted feature assessments and extractions to prevent external bias. To determine the heterogeneity in feature assessments between the two neurosurgeons, we developed a heatmap of features and measured the Kappa coefficient to determine the consistency of feature assessments. The Kappa coefficients for all variables were greater than 0.78 (*P* < 1.0 × 10^− 4^), demonstrating excellent inter-rater agreement and supporting the validity of the current indicator.

### MRI acquisition

MRI data were obtained in the diagnostic room of an MRI-integrated neurosurgical suite (IMRIS, Winnipeg, Canada) using a 3.0T scanner (MAGNETOM Verio 3.0T; Siemens AG, Erlangen, Germany). A fluid-attenuated inversion recovery (FLAIR) sequence was used for structural imaging (TR, 9000 ms; TE, 96 ms; TI, 2500 ms; flip angle, 150 degrees; slice thickness, 2 mm; field of view [FOV], 240 × 240 mm; matrix size, 256 × 160). The T1-weighted magnetization prepared rapid gradient echo (MPRAGE) image was used for feature extraction (TR, 1900 ms; TE, 2.93 ms; flip angle, 9 degrees; matrix size, 256 × 215; slice number, 176; slice thickness, 1 mm; FOV, 250 × 219 mm; acquisition average, 1; scanning time, 7 min 49 s). Gadolinium (GE Healthcare, Cork, Ireland) was used as a MRI contrast agent for contrast-enhanced T1-weighted imaging. MRI brain scans were obtained within 3 days before surgery for each participant.

### Determination of molecular markers

MGMT promotor methylation analysis was performed via methylation-specific polymerase chain reaction (PCR). 1p/19q codeletion was determined by fluorescence in situ hybridization (FISH) or shallow whole-genome sequencing (sWGS). IDH1/2 and TERT promoter mutational status were detected via Sanger sequencing, covering codon 132 of IDH1 and codon 172 of IDH2, C228T, and C250T in the TERT core promoter. IDH status in every mutant case was confirmed by independent PCR amplification and sequencing.

### Statistical analysis

Figure S1 is a technology roadmap showing the process steps of this study. In the cohort database, we used RPA to explore the joint effects of multiple molecular markers on the survival of glioma patients, developed survival groups via partDSA algorithm, and displayed the groupings as a survival tree. The corresponding Kaplan-Meier survival curve for each group was plotted, and the log-rank test was used to evaluate the survival differences between the groups.

In the imaging database, we used clinical information and VASARI features to make predictions for the molecular markers contributing to the survival risk grouping. Differences in chief complaints, onset symptoms, and VASARI feature set between the groups varying in molecular marker status were compared by t-test or chi-square test. Variables with p-values less than 0.1 in univariate analysis were selected for multivariate analysis for constructing predictive models of molecular marker status. Multivariate analysis used logistic regression models to evaluate the association of potential predictors with molecular marker status. Following the results of multivariate analysis, the receiver operating characteristic (ROC) curves were generated to evaluate the performance of molecular marker prediction in terms of area under the curve (AUC) value. Nomograms were developed as a scoring prediction system for molecular markers to facilitate clinical application. The logistic multivariate regression model was used to generate a nomogram based on the regression coefficients of the predictors in the multivariate model, scoring each indicator at different levels and totaling these scores. Subsequently, the likelihood of mutation of the molecular markers was estimated using the functional transformation between the total score and the probability of occurrence in the regression model. We employed an independent database from The Cancer Genome Atlas (TCGA) to externally validate the IDH prediction model, as presented in a distinct manuscript. However, the external validation of the TERT prediction model was not feasible due to the absence of TERT mutation data within the validation dataset. We performed an internal validation of the TERT mutation prediction model using Bootstrap resampling, setting up with playback resampling 1,000 times, plotting the ROC curves obtained from each resampling, and calculating the average AUC values for the 1,000 times, denoted as mean-AUC. All analyses were performed with SAS v.9.4 and R v.4.3.2.

## Results

### Patient characteristics

The cohort dataset comprised 238 patients, with a mean age of 47.81 years. Of these patients, a majority were male (131; 55.04%), while females accounted for 44.96% (107 patients) as shown in Table 1. A significant portion of the cohort presented with IDH mutations (131/238; 55.04%). TERT promoter mutations were observed in 46.64% (111/238), MGMT promoter methylation in 36.55% (87/238), and 1p/19q codeletion in 34.03% (81/238) of patients. The imaging dataset included 113 subjects with a mean age of 44.35 years (IQR: 36.00–53.00 years), consisting of 60 men (53.10%) and 53 women (46.90%). IDH mutations were prevalent in 58.41% (66/113) of the subjects, while TERT promoter mutations were found in 38.05% (43/113). Notably, a high proportion of subjects (98/113; 86.73%) maintained an independent Karnofsky Performance Status (KPS) score of 90. Comprehensive clinical details for the subjects in the imaging dataset are provided in Table 1.

**Table 1:** Patients characteristics

Characteristic	Cohort Dataset (*n* = 238)	Imaging Dataset (*n* = 113)
Age (years)		
Mean (SD)	47.81 (13.16)	44.35 (11.18)
Median (IQR)	47.00 (39.00–57.00)	45.00 (36.00–53.00)
Range	7.00-119.00	17.00–68.00
Gender		
Male (%)	131 (55.04)	60 (53.10)
Female (%)	107 (44.96)	53 (46.90)
Illness Duration (months)		
Mean (SD)	6.08 (15.07)	12.15 (31.03)
Median (IQR)	1.00 (0.50-4.00)	2.00 (1.00–6.00)
Range	0.10–120	0.10–240.00
IDH status		
Wild Type (%)	107 (44.96)	47 (41.59)
Mutant (%)	131 (55.04)	66 (58.41)
TERT promoter status		
Wild Type (%)	127 (53.36)	65 (57.52)
Mutant (%)	111 (46.64)	43 (38.05)
Unknown (%)	0 (0)	5 (4.42)
MGMT status		
Methylated (%)	87 (36.55)	40 (35.40)
Unmethylated (%)	151 (63.45)	66 (58.41)
Unknown (%)	0 (0)	7 (6.19)
1p/19q status		
Codeletion (%)	81 (34.03)	21 (18.58)
Intact (%)	157 (65.97)	78 (69.03)
Unknown (%)	0 (0)	14 (12.39)
Survival status		
Censored (%)	180 (75.63)	91 (80.53)
Dead (%)	58 (24.37)	22 (19.47)
Preoperative KPS		
< 60 (%)	2 (0.84)	1 (0.88)
60 (%)	3 (1.26)	0 (0)
70 (%)	1 (0.42)	1 (0.88)
80 (%)	14 (5.88)	12 (10.62)
90 (%)	217 (91.18)	98 (86.73)
100 (%)	1 (0.42)	1 (0.88)
Median KPS (IQR)	90 (90–90)	90 (90–90)
Sudden Disturbance or Loss of Consciousness		
Yes (%)	-	28 (24.78)
No (%)	-	85 (75.22)
Dizziness		
Yes (%)	-	15 (13.27)
No (%)	-	98 (86.73)
Headache (Chief Complaint)		
Yes (%)	-	35 (30.97)
No (%)	-	78 (69.03)
Limb Twitching		
Yes (%)	-	28 (24.78)
No (%)	-	85 (75.22)
Limb Numbness		
Yes (%)	-	14 (12.39)
No (%)	-	99 (87.61)
Seizures (Chief Complaint)		
Yes (%)	-	10 (8.85)
No (%)	-	103 (91.15)
Speech Vague		
Yes (%)	-	6 (5.31)
No (%)	-	107 (94.69)
Epilepsy		
Yes (%)	-	43 (38.05)
No (%)	-	70 (61.95)
Headache (Onset Symptom)		
Yes (%)	-	35 (30.97)
No (%)	-	78 (69.03)
Speech Impairment		
Yes (%)	-	13 (11.50)
No (%)	-	100 (88.50)
Visual Impairment		
Yes (%)	-	24 (21.24)
No (%)	-	89 (78.76)

Abbreviations: IDH, isocitrate dehydrogenase; TERT, telomerase reverse transcriptase; MGMT, O6-methylguanine-DNA methyltransferase; IQR, interquartile range; KPS, Karnofsky Performance Score

### Identification of Survival Risk groups

**Fig. 2:**
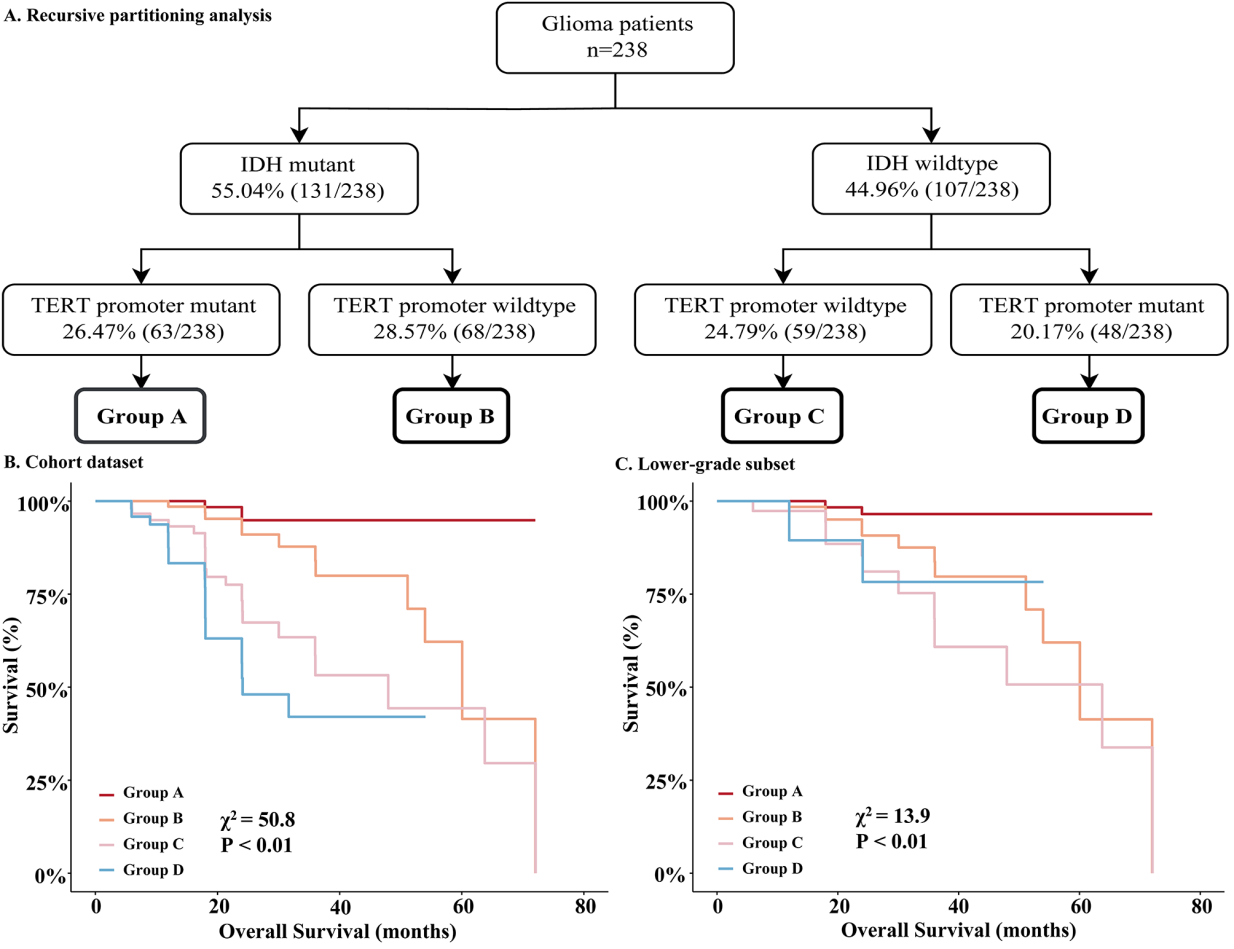
Recursive partitioning analysis (RPA) and survival analysis. The recursive partitioning analysis (RPA) identified four distinct survival risk groups using IDH and TERT mutation status **(A)**. Group A comprised 26.47% (63/238) of patients harboring mutations in both IDH and TERT promoters. Group B included 28.57% (68/238) with IDH mutations but wildtype TERT. Group C contained 24.79% (59/238) of patients with wildtype IDH and wildtype TERT, while Group D, with 20.17% (48/238), was characterized by wildtype IDH and mutant TERT. Kaplan-Meier survival curves for these groups are shown in **(B)** for the entire cohort and **(C)** for the lower-grade glioma subset. Patients with IDH mutations (Groups A and B, depicted in red and orange) displayed longer survival compared to IDH wildtype patients (Groups C and D, shown in pink and blue). The presence of TERT mutations conferred a survival benefit in IDH mutant gliomas but was associated with poorer outcomes in IDH wildtype gliomas. Log-rank tests revealed significant survival differences among the risk groups within the full cohort (χ2 = 50.8, *P* < 0.01) and the lower-grade subset (χ2 = 13.9, *P* < 0.01)

Figure 2A and B showed how IDH and TERT can classify patients with gliomas into four groups with significant differences in survival. The Group A, those having mutations in both the IDH and TERT promoters, was the best in terms of survival. Group B contained individuals with IDH mutations but no mutations in the TERT promoter, and survival was marginally poorer in group B than in group A. Group C patients had worse overall survival than patients in group B, with no mutations in either IDH or TERT promoter. Patients with IDH wildtype (IDHwt) and TERTmut in Group D exhibited the worst overall survival compared to the preceding three groups. Additionally, we extracted patients of specific grades from the cohort dataset to form two separate subsets and used the aforementioned risk classification to plot Kaplan-Meier curves in different subsets (Fig. 2C and Fig. S2 in supplementary).

### VASARI Imaging features predicting IDH and TERT promoter status

**Fig. 3:**
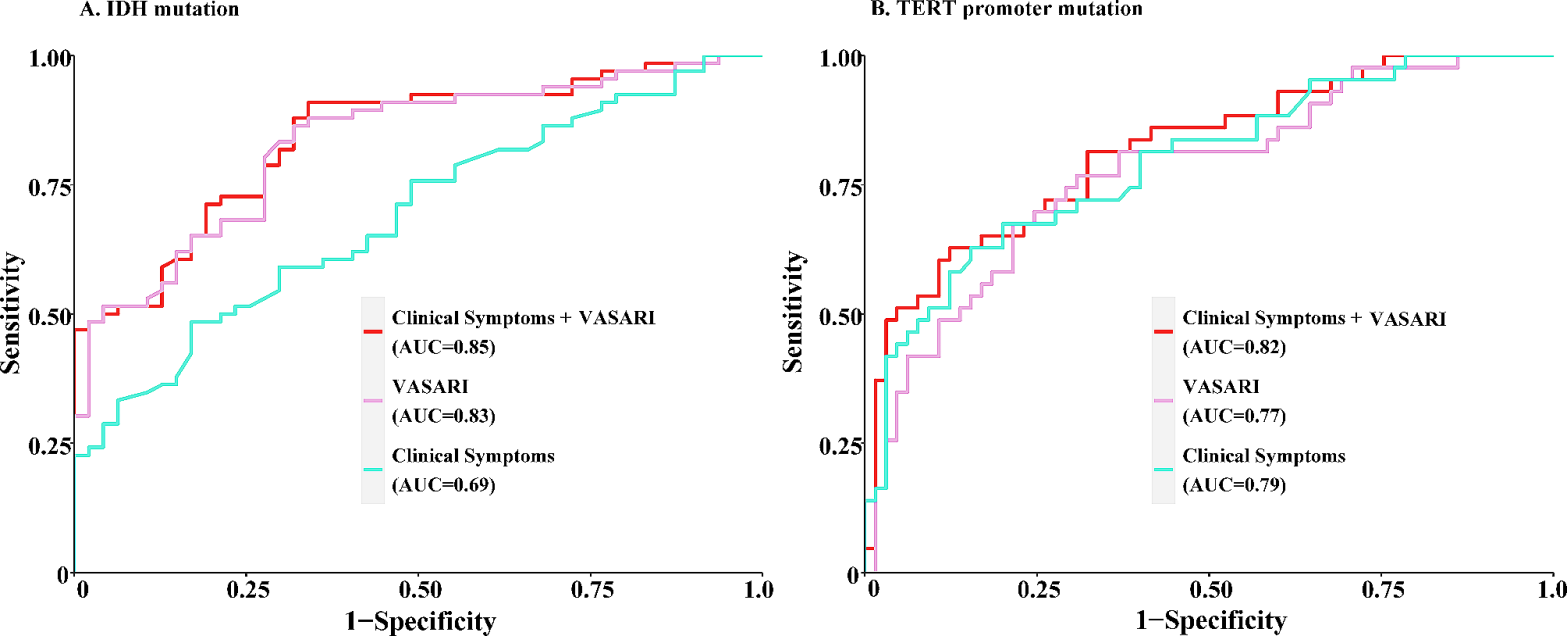
ROC Curves for Predicted IDH and TERT mutations based on VASARI features and clinical symptoms. ROC curves display the predictive accuracy for IDH **(A)** and TERT promoter mutations **(B)** utilizing multiple logistic regression models. The integration of VASARI MRI features and clinical symptoms yielded AUC values of 0.85 for IDH and 0.82 for TERT mutations, demonstrating enhanced predictive performance compared to using either VASARI MRI features or clinical symptoms independently

Based on the survival risk grouping findings derived by RPA, we further employed the imaging features to predict IDH and TERT status. Pial invasion and non-enhancing margin were important predictors of IDH mutation (Table S1 and Table S2 in the Supplementary). The ROC curves (Fig. 3A) indicated the best predictive performance of VASARI features paired with clinical symptoms (chief complaints and onset symptoms) for IDH prediction with an AUC value of 0.85, compared to 0.83 and 0.69 for VASARI imaging features or clinical symptoms alone. The predictive accuracy of the IDH prediction model in the TCGA dataset was statistically significant form 50% (Z = 2.11, *P* = 0.04) and the predicted IDH status obtained in TCGA still significantly differentiated glioma patient survival (χ2 = 4.90, *P* = 0.03, Fig. S3).

Imaging data from 108 subjects were used for the prediction of TERT promoter mutation status, of which 43 (39.81%) were TERTmut and 65 (60.19%) were TERT wildtype (TERTwt) (Table S3 in Supplementary). TERTmut patients were significantly older than TERTwt patients (mean [SD] age, 48.07 [11.36] vs. 42.32 [10.69] years, t = -2.67, *P* = 0.01). Individuals with TERT mutations in gliomas were more likely to develop epilepsy (χ^2^ = 6.41, *P* = 0.01) or visual impairment (χ^2^ = 4.28, *P* = 0.04) as their onset symptoms. TERTwt tumors were more likely to involve the speech motor area (χ^2^ = 4.65, *P* = 0.03) and the non-enhancing margin was smoother (χ^2^ = 6.26, *P* = 0.01) compared to TERTmut tumors. There were also significant differences in tumor location (χ^2^ = 4.02, *P* = 0.04) and side of tumor epicenter (Z = -2.17, *P* = 0.03) between TERTmut and TERTwt groups. Age (odds ratio [OR] = 1.06, *P* = 0.01) and epilepsy (OR = 5.73, *P* = 0.03) were revealed to be important predictors of TERT mutation in the subsequent multivariate logistic regression model (Table S4 in the Supplementary). In the ROC curve analysis (Fig. 3B), the AUC value for predicting TERT mutations using VASARI and clinical symptoms was 0.82. The data were resampled 1000 times using the Bootstrap method, and ROC curves were plotted for each resampled sample using the predictive model (Fig. S4), and the mean AUC (mean-AUC) for resampling remained 0.82, with a 95% confidence interval of (0.819, 0.824) for mean-AUC.

**Fig. 4:**
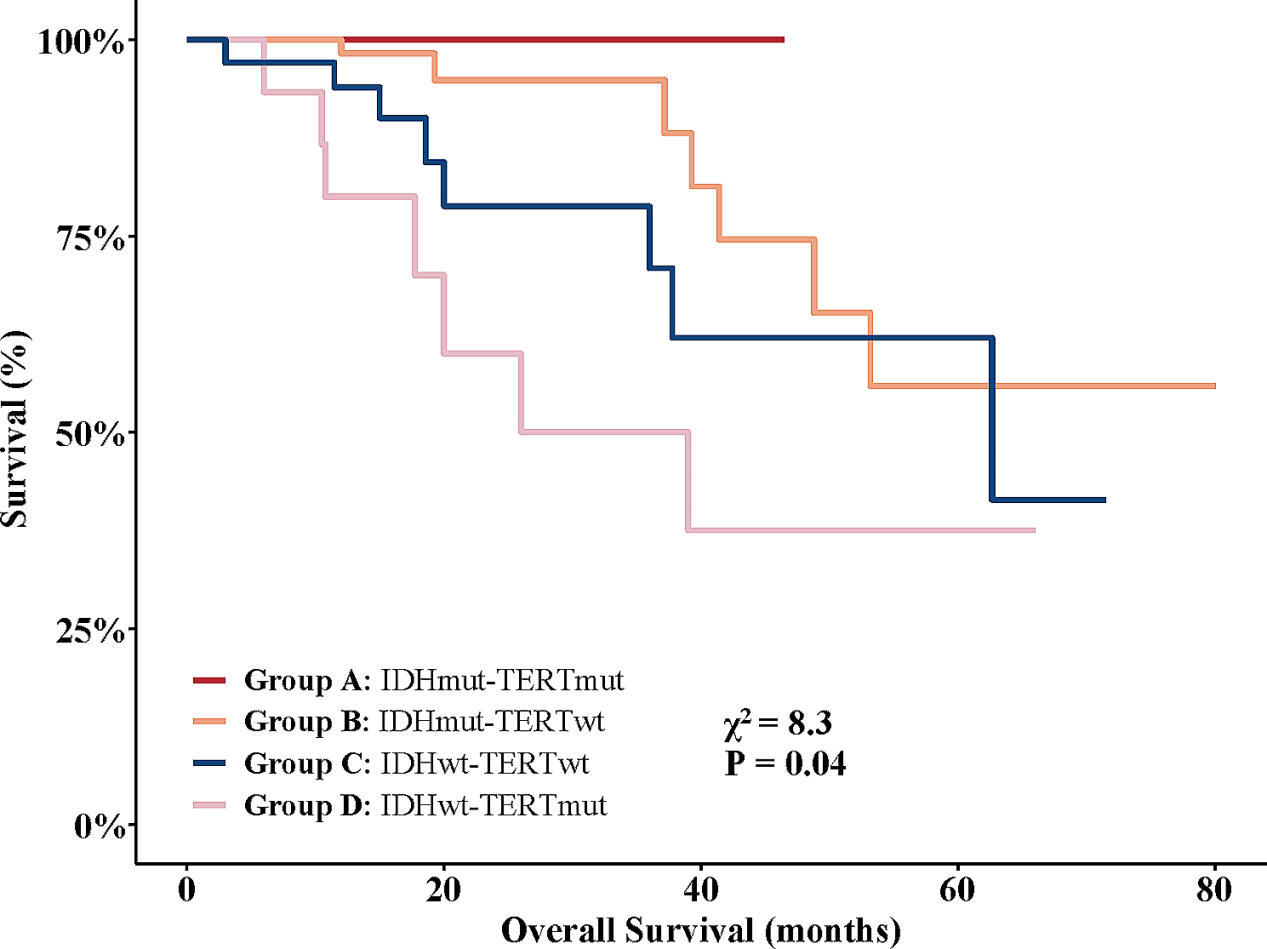
Kaplan-Meier survival estimates for risk groups based on imaging features. Patients predicted of IDH/TERT status using VASARI imaging features showed a significant difference in survival was observed across the predicted groups (Log-rank test, χ² = 8.3, *P* = 0.04), with Group A displaying the most favorable prognosis and Group D the least

The survival analysis of the VASARI predicted risk groupings was shown in Fig. 4. The cutoff level of IDH was the closest point to the upper left corner based on ROC analysis. Since TERT mutation is an unfavorable factor in IDHwt tumors, once a patient with glioma is predicted to be IDHwt-TERTmut, it may demand sufficient attention from physicians and impose additional psychological and testing expense burden on the patient. To decrease the possibility that individuals with TERTwt glioma are wrongly predicted as TERTmut, we changed the cutoff value to reach a specificity of at least 90% for TERT mutant prediction. Figure 4 exhibited a similar survival distribution to Fig. 2B, with the IDHmut-TERTmut group having the best survival and the IDHwt-TERTmut group showing the worst survival.

### Individualized scoring tools based on nomogram models

**Fig. 5:**
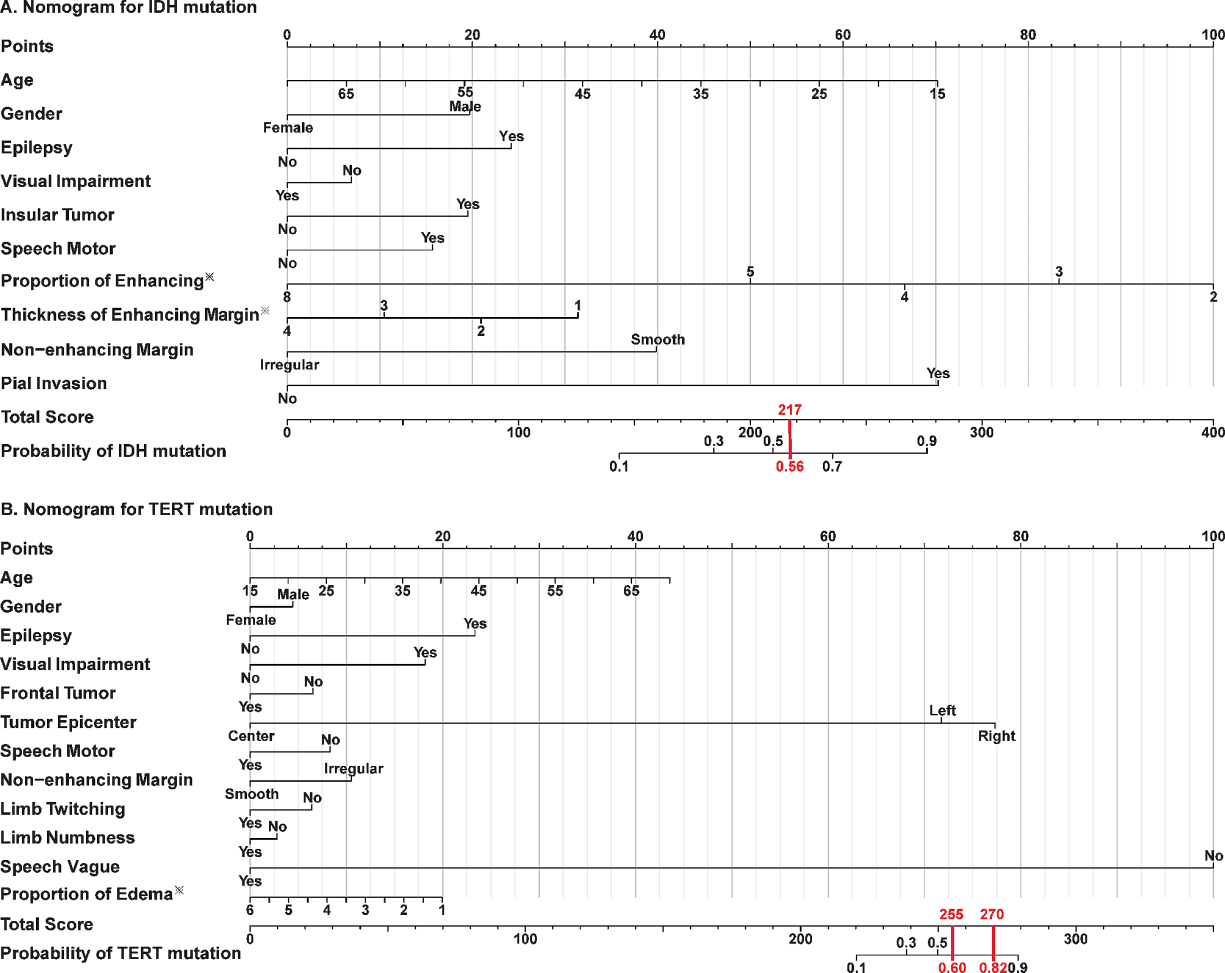
Nomograms for prediction of IDH and TERT mutations in glioma patients. Nomograms were developed from logistic regression models to estimate the probability of IDH mutation and TERT mutations in glioma patients. The total score, derived from individual clinical and radiological features, predicts the likelihood of an IDH/TERT mutation. **(A)** A total score exceeding 217 suggests a high probability of IDH mutation presence. **(B)** For patients with IDH mutation (IDHmut), a total score over 270, and for those without IDH mutation (IDHwt), a score over 255, indicates a significant likelihood of TERT mutation. **Key Definitions**: Speech Motor Involvement: Reflects whether the glioma involves eloquent cortical areas or crucial subcortical white matter related to motor function. Non-enhancing Margin: The outer non-enhancing margin of the tumor is categorized as either well-defined (smooth) or ill-defined (irregular). Pial Invasion: Denotes the presence of enhancement in the pial layers contiguous with the enhancing or non-enhancing tumor mass. Tumor Epicenter: The location of the central mass of the lesion, classified as left, center, or right. **Note**^※^: Proportions of enhancing and edema are classified on a scale where values 2 to 8 represent percentages as follows: 0%, < 5%, 6–33%, 34–67%, and 100%. Similarly, the thickness of the enhancing margin is rated from 1 to 4, indicating not applicable, none, thin, and thick. These variables and their implications for mutation prediction are detailed in the supplementary material

We developed nomogram models, depicted in Fig. 5, which combine VASARI features and clinical indicators to serve as an individualized scoring tool for predicting IDH and TERT promoter mutations, based on the outcomes of multivariate logistic regression analysis. As shown in Fig. 5, each variable option corresponds to a score on the first horizontal axis, denoted as points. The total scores are calculated by summing the scores of a patient’s characteristics, with the probability of an IDH or TERT promoter mutation (displayed on the final horizontal axis) being derived from the corresponding total score. Notably, males and epilepsy are prevalent factors that increase the likelihood of mutations in both IDH and TERT promoters. In Fig. 5A, other variables that enhance the probability of IDH mutations include younger age, absence of visual impairment, insular lobe tumor, speech motor area involved, low proportional of enhancement areas, thin enhancement margins, smooth non-enhancing margins, and pial invasion. However, TERT promoter mutations are more likely to be observed in patients with older age, visual impairment, non-frontal lobe tumors, speech motor area not involved, right-sided tumor epicenter, irregular non-enhancing margins, no limb twitching or limb numbness, no speech vague, and a low proportion of edema. Patients with a total score greater than 217 were expected to have a mutation in IDH. IDHmut patients with a total score larger than 270 were expected to be TERTmut, while IDHwt patients with a total score greater than 255 were predicted to have a TERT mutation.

## Discussion

In this study, we gathered a large comprehensive cohort dataset and an imaging dataset to construct survival risk groups and predict molecular metrics, respectively. Among MGMT, IDH, TERT, and 1p/19q, we identified the most important molecular biomarker combination associated with patients’ overall survival. People with IDHmut-TERTmut glioma exhibited the best overall survival, while those with IDHwt-TERTmut glioma had the poorest. Moreover, using Visually Accessible Rembrandt Images (VASARI) imaging features along with the clinical symptoms, the AUC values for predicting such a combination reached a high value of 0.85 and 0.82, respectively. Corresponding nomograms have also been developed as a clinical scoring system for predicting molecular indicators preoperatively.

In our study, Recursive Partitioning Analysis (RPA) was used to analyze the prognosis of glioma patients by considering a range of molecular markers and clinical variables. Grouping by IDH and TERT status emerged as a significant method for distinguishing survival outcomes. IDH and TERT proved to be the most influential markers in classifying patients into distinctly different survival subgroups. For lower-grade gliomas, the coexistence of IDH and TERT mutations was associated with a survival advantage, corroborating existing literature [28]. Contrary to prior findings that TERT mutations are linked with poor prognosis in glioblastoma [27, 28], our analysis did not indicate a detrimental impact of TERT mutations on survival in such cases. The complex interplay between cell transformation, the tumor microenvironment, and inflammation, particularly neutrophil infiltration related to TERT mutations, may underlie the challenging prognosis of IDHwt gliomas [29–32].

The relationship between molecular biomarkers and clinical variables in glioma survival prediction is complex, especially when multiple biomarkers are present, and a clear framework to interpret these interactions is not established [20, 33, 34]. While COX regression is a commonly used method for survival analysis, it has limitations, including the need to verify the proportional hazards assumption and to understand potential interactions between variables [13, 35]. Recursive Partitioning Analysis (RPA) offers an alternative that is well-suited for censored data and avoids the pitfalls of overfitting [18, 19]. It is effective in categorizing glioma survival risk and offers a pragmatic approach for creating clinically relevant risk stratification tools. Our research advances beyond prior studies that have delineated survival distributions within particular molecular subgroups of glioma patients [36, 37]. We sought to refine subgrouping by employing algorithms to generate more homogeneously defined risk groups based on survival outcomes. Unlike studies that have manually classified patients based solely on IDH and TERT status [24], our methodology dynamically integrates multiple variables to identify the most prognostically significant subgroups. This algorithmic approach allows for a more nuanced assessment of glioma prognosis, moving away from the conventional practice of manually selecting subsequent markers following IDH, thus striving for a more data-driven determination of clinically relevant subgroups.

Using VASARI, we were able to predict molecular markers preoperatively in order to predict patients’ survival. The superior predictive performance for IDH mutation, signified by an AUC of 0.85 when integrating VASARI with clinical symptoms, underscores the synergy between imaging and symptomatic data. This enhances the preoperative stratification process, potentially refining surgical planning and adjuvant therapy decisions. The association of TERT mutations with older age and particular onset symptoms like epilepsy or visual impairment hints at a more nuanced understanding of glioma biology [38, 39]. Notably, the multivariate analysis further reinforced the importance of age and epilepsy as predictors of TERT mutation status. The utility of such multivariate models is embodied in the development of our nomograms, which offer a quantifiable means to predict molecular mutations. These tools bridge the gap between clinical and imaging assessments, providing a tailored approach to patient prognosis. These findings not only validate the utility of VASARI imaging as a non-invasive tool for preoperative molecular profiling [40] but also highlight the potential of incorporating clinical indicators to refine this predictive capacity.

VASARI imaging features have been applied in prior research to construct predictive models [11, 23, 41]. The need for increasingly larger patient groups as more molecular markers are considered often restricts the analysis. Moreover, these studies have not fully addressed how to synthesize information from multiple molecular markers to inform prognosis. This is a critical gap, as a single patient may present with multiple markers that could have conflicting impacts on survival. For instance, the prognostic implications of an IDHmut glioma with both 1p/19q codeletion, which is beneficial, and TERT wildtype, which is not, raises the question of which markers or combinations thereof most significantly affect survival [12, 28]. To address this, our study utilized the RPA algorithm to develop a framework for survival risk grouping prior to molecular marker prediction, aiming to identify the most consequential combinations for patient survival. The algorithm selectively identified the combination of IDH and TERT mutations as having the most significant impact on survival. By applying this framework and integrating VASARI imaging features, we aim to enhance the ability of physicians to make informed survival assessments, taking into account a comprehensive array of preoperative molecular data. Our objective was to leverage preoperative molecular markers to simultaneously achieve survival prediction and guidance of surgical strategy. Therefore, on the basis of the algorithm identifying the molecular markers that best distinguish survival, we considered utilizing all easily accessible and bio-interpretable common preoperative features, such as basic information and chief complaints, and of course MRI images to help obtain preoperative molecular marker information. In this way, preoperative data can be used to simultaneously determine postoperative survival and improve postoperative survival quality by guiding surgical strategy.

Various studies have indicated that the benefit of surgical resection is influenced by the status of molecular markers. Previous studies have shown that IDH and TERT double mutations often occur simultaneously with 1p / 19q codeletion in adult gliomas [9], and such gliomas are classified as oligodendroglioma according to the 2021 version of the CNS classification criteria. For TERTmut oligodendroglioma, a small residual has little impact on the patient’s prognosis [25]. Studies indicate that for TERTwt astrocytomas, expanding the resection range can greatly enhance patients’ prognoses and that the total resection of imaging borders is a suitable approach [26, 42]. Our previous study [43] and another relevant study [44] found that a surgical strategy of resection of enhancing and T2/FLAIR high signal areas (non-enhancing areas) is viable for IDHwt gliomas that meet the diagnostic criteria for glioblastoma. In clinical practice, the goal of surgery is to maximize tumor resection within functional boundaries as much as possible, and this does not change depending on predicted molecular marker status. Conversely, for predicted molecular markers status that is unfavorable for survival, the predicted results may serve as a basis to support extensive resection. In addition, preoperative information about the nature of the glioma can help to more fully assess and discuss the need for a temporary deficit as a result of surgery. This not only provides a scientific basis for the development of a surgical strategy but also facilitates informed decision-making and communication between the patient and the surgeon during preoperative discussions.

This study has several limitations. Firstly, our study’s sample sizes for the RPA and imaging datasets were reduced due to the requirement of complete information on four molecular markers. The evolving landscape of molecular marker discovery and financial constraints limited access to comprehensive molecular profiling. Despite efforts to incorporate relevant markers, the high costs of molecular testing remain a barrier. Our study highlights the need for preoperative noninvasive methods to provide prognostic insights without extensive molecular testing. Future studies with larger cohorts and more comprehensive molecular marker data are necessary to validate and refine our findings. Secondly, the study’s retrospective design precluded the ability to control for potential confounding variables or implement standardized data collection protocols. Moreover, physician preferences in decision-making regarding patient medication and surgical interventions may have introduced biases into the analysis. To enhance the robustness of future research, we recommend collecting prospective data from multiple institutions. This approach would mitigate potential biases and enrich the breadth and depth of insights gained from the study, ultimately strengthening the generalizability of the findings. Finally, while VASARI features extracted from the two MRI sequences (T1-weighted and Flair) have shown good predictive value, the inclusion of additional MRI sequences such as MR Spectroscopy, Diffusion Imaging, etc., may further improve the accuracy of molecular marker prediction in the future.

## Conclusion

In this study, we utilized multiple molecular markers and non-invasive VASARI imaging features to determine the survival prognosis of glioma patients and guide treatment strategy. Using the recursive partitioning survival analysis, the combination of IDH and TERT was found to successfully classify gliomas into risk subgroups with significant differences in survival. Furthermore, an individualized nomogram prediction tool was developed for the prediction of IDH and TERT mutations. These findings offer insights into the development of a more personalized approach to glioma treatment, with the potential to improve patient outcomes.

## Electronic supplementary material

Below is the link to the electronic supplementary material.

Supplementary Material 1

## Data Availability

Anonymized data not published within this article will be made available by request from any qualified investigator.

## Abbreviations

IDH: Isocitrate dehydrogenase
TERT: Telomerase reverse transcriptase
MGMT: O6-methylguanine-DNA methyltransferase
VASARI: Visually Accessible Rembrandt Images
RPA: Recursive partitioning analysis
ROC: Receiver operator characteristic
AUC: Area under the receiver operating characteristic curve
WHO: World Health Organization
MRI: Magnetic resonance imaging

## References

[1] Rasmussen BK, Hansen S, Laursen RJ, Kosteljanetz M, Schultz H, Norgard BM, Guldberg R, Gradel KO. Epidemiology of glioma: clinical characteristics, symptoms, and predictors of glioma patients grade I-IV in the the Danish Neuro-Oncology Registry. J Neurooncol. 2017;135(3):571–9. 28861666 10.1007/s11060-017-2607-5

[2] Nabors LB, Portnow J, Ammirati M, Baehring J, Brem H, Butowski N, Fenstermaker RA, Forsyth P, Hattangadi-Gluth J, Holdhoff M, et al. NCCN guidelines insights: Central Nervous System Cancers, Version 1.2017. J Natl Compr Canc Netw. 2017;15(11):1331–45. 29118226 10.6004/jnccn.2017.0166

[3] Brown TJ, Brennan MC, Li M, Church EW, Brandmeir NJ, Rakszawski KL, Patel AS, Rizk EB, Suki D, Sawaya R, et al. Association of the extent of Resection with Survival in Glioblastoma: a systematic review and Meta-analysis. JAMA Oncol. 2016;2(11):1460–9. 27310651 10.1001/jamaoncol.2016.1373 PMC6438173

[4] Ohgaki H, Kleihues P. Epidemiology and etiology of gliomas. Acta Neuropathol. 2005;109(1):93–108. 15685439 10.1007/s00401-005-0991-y

[5] Ostrom QT, Bauchet L, Davis FG, Deltour I, Fisher JL, Langer CE, Pekmezci M, Schwartzbaum JA, Turner MC, Walsh KM, et al. The epidemiology of glioma in adults: a state of the science review. Neuro Oncol. 2014;16(7):896–913. 24842956 10.1093/neuonc/nou087 PMC4057143

[6] Stupp R, Hegi ME, Mason WP, van den Bent MJ, Taphoorn MJ, Janzer RC, Ludwin SK, Allgeier A, Fisher B, Belanger K, et al. Effects of radiotherapy with concomitant and adjuvant temozolomide versus radiotherapy alone on survival in glioblastoma in a randomised phase III study: 5-year analysis of the EORTC-NCIC trial. Lancet Oncol. 2009;10(5):459–66. 19269895 10.1016/S1470-2045(09)70025-7

[7] Molinaro AM, Taylor JW, Wiencke JK, Wrensch MR. Genetic and molecular epidemiology of adult diffuse glioma. Nat Rev Neurol. 2019;15(7):405–17. 31227792 10.1038/s41582-019-0220-2 PMC7286557

[8] Louis DN, Perry A, Wesseling P, Brat DJ, Cree IA, Figarella-Branger D, Hawkins C, Ng HK, Pfister SM, Reifenberger G, et al. The 2021 WHO classification of tumors of the Central Nervous System: a summary. Neuro Oncol. 2021;23(8):1231–51. 34185076 10.1093/neuonc/noab106 PMC8328013

[9] Eckel-Passow JE, Lachance DH, Molinaro AM, Walsh KM, Decker PA, Sicotte H, Pekmezci M, Rice T, Kosel ML, Smirnov IV, et al. Glioma groups based on 1p/19q, IDH, and TERT promoter mutations in tumors. N Engl J Med. 2015;372(26):2499–508. 26061753 10.1056/NEJMoa1407279 PMC4489704

[10] Mizoguchi M, Hata N, Kuga D, Hatae R, Akagi Y, Sangatsuda Y, Fujioka Y, Takigawa K, Funakoshi Y, Suzuki SO, et al. Clinical implications of molecular analysis in diffuse glioma stratification. Brain Tumor Pathol. 2021;38(3):210–7. 34268651 10.1007/s10014-021-00409-y

[11] Hyare H, Rice L, Thust S, Nachev P, Jha A, Milic M, Brandner S, Rees J. Modelling MR and clinical features in grade II/III astrocytomas to predict IDH mutation status. Eur J Radiol. 2019;114:120–7. 31005161 10.1016/j.ejrad.2019.03.003

[12] Labussiere M, Idbaih A, Wang XW, Marie Y, Boisselier B, Falet C, Paris S, Laffaire J, Carpentier C, Criniere E, et al. All the 1p19q codeleted gliomas are mutated on IDH1 or IDH2. Neurology. 2010;74(23):1886–90. 20427748 10.1212/WNL.0b013e3181e1cf3a

[13] Zhou Y, McArdle JJ. Rationale and applications of Survival Tree and Survival Ensemble methods. Psychometrika. 2015;80(3):811–33. 25228495 10.1007/s11336-014-9413-1 PMC4409541

[14] Basak P, Linero A, Sinha D, Lipsitz S. Semiparametric analysis of clustered interval-censored survival data using soft bayesian additive regression trees (SBART). Biometrics. 2022;78(3):880–93. 33864633 10.1111/biom.13478

[15] Linero AR, Basak P, Li Y, Sinha D. Bayesian survival tree ensembles with Submodel Shrinkage. Bayesian Anal 2022, 17(3).

[16] Hothorn T, Buhlmann P, Dudoit S, Molinaro A, van der Laan MJ. Survival ensembles. Biostatistics. 2006;7(3):355–73. 16344280 10.1093/biostatistics/kxj011

[17] Safiyari A. R Javidan 2017 Predicting lung cancer survivability using ensemble learning methods. Intell Syst Conf (IntelliSys) 2017 684–8.

[18] Molinaro AM, Lostritto K, van der Laan M. partDSA: deletion/substitution/addition algorithm for partitioning the covariate space in prediction. Bioinformatics. 2010;26(10):1357–63. 20375111 10.1093/bioinformatics/btq142 PMC2865863

[19] Lostritto K, Strawderman RL, Molinaro AM. A partitioning Deletion/Substitution/Addition algorithm for creating survival risk groups. Biometrics. 2012;68(4):1146–56. 22519965 10.1111/j.1541-0420.2012.01756.x

[20] Wiencke JK, Zhang Z, Koestler DC, Salas LA, Molinaro AM, Christensen BC, Kelsey KT. Identification of a foetal epigenetic compartment in adult human kidney. Epigenetics. 2022;17(3):335–55. 33783321 10.1080/15592294.2021.1900027 PMC8920245

[21] Audureau E, Chivet A, Ursu R, Corns R, Metellus P, Noel G, Zouaoui S, Guyotat J, Le Reste P-J, Faillot T, et al. Prognostic factors for survival in adult patients with recurrent glioblastoma: a decision-tree-based model. J Neurooncol. 2018;136(3):565–76. 29159777 10.1007/s11060-017-2685-4

[22] Molinaro AM, Hervey-Jumper S, Morshed RA, Young J, Han SJ, Chunduru P, Zhang Y, Phillips JJ, Shai A, Lafontaine M, et al. Association of Maximal Extent of Resection of contrast-enhanced and non-contrast-enhanced Tumor with Survival within Molecular subgroups of patients with newly diagnosed Glioblastoma. JAMA Oncol. 2020;6(4):495–503. 32027343 10.1001/jamaoncol.2019.6143 PMC7042822

[23] Park YW, Han K, Ahn SS, Bae S, Choi YS, Chang JH, Kim SH, Kang SG, Lee SK. Prediction of IDH1-Mutation and 1p/19q-Codeletion status using preoperative MR Imaging Phenotypes in Lower Grade Gliomas. AJNR Am J Neuroradiol. 2018;39(1):37–42. 29122763 10.3174/ajnr.A5421 PMC7410710

[24] Yang P, Cai J, Yan W, Zhang W, Wang Y, Chen B, Li G, Li S, Wu C, Yao K, et al. Classification based on mutations of TERT promoter and IDH characterizes subtypes in grade II/III gliomas. Neuro Oncol. 2016;18(8):1099–108. 26957363 10.1093/neuonc/now021 PMC4933482

[25] Delev D, Heiland DH, Franco P, Reinacher P, Mader I, Staszewski O, Lassmann S, Grau S, Schnell O. Surgical management of lower-grade glioma in the spotlight of the 2016 WHO classification system. J Neurooncol. 2019;141(1):223–33. 30467813 10.1007/s11060-018-03030-w

[26] Wijnenga MMJ, French PJ, Dubbink HJ, Dinjens WNM, Atmodimedjo PN, Kros JM, Smits M, Gahrmann R, Rutten GJ, Verheul JB, et al. The impact of surgery in molecularly defined low-grade glioma: an integrated clinical, radiological, and molecular analysis. Neuro Oncol. 2018;20(1):103–12. 29016833 10.1093/neuonc/nox176 PMC5761503

[27] Cancer Genome Atlas, Research N, Brat DJ, Verhaak RG, Aldape KD, Yung WK, Salama SR, Cooper LA, Rheinbay E, Miller CR, Vitucci M, et al. Comprehensive, Integrative Genomic Analysis of Diffuse Lower-Grade Gliomas. N Engl J Med. 2015;372(26):2481–98. 26061751 10.1056/NEJMoa1402121 PMC4530011

[28] Vuong HG, Altibi AMA, Duong UNP, Ngo HTT, Pham TQ, Chan AK, Park CK, Fung KM, Hassell L. TERT promoter mutation and its interaction with IDH mutations in glioma: combined TERT promoter and IDH mutations stratifies lower-grade glioma into distinct survival subgroups-A meta-analysis of aggregate data. Crit Rev Oncol Hematol. 2017;120:1–9. 29198322 10.1016/j.critrevonc.2017.09.013

[29] Hewedi IH, Radwan NA, Shash LS, Elserry TH. Perspectives on the immunologic microenvironment of astrocytomas. Cancer Manag Res. 2013;5:293–9. 24039448 10.2147/CMAR.S48942 PMC3770516

[30] Poon CC, Sarkar S, Yong VW, Kelly JJP. Glioblastoma-associated microglia and macrophages: targets for therapies to improve prognosis. Brain. 2017;140(6):1548–60. 28334886 10.1093/brain/aww355

[31] Mantovani A, Allavena P, Sica A, Balkwill F. Cancer-related inflammation. Nature. 2008;454(7203):436–44. 18650914 10.1038/nature07205

[32] Hou Z, Zhang K, Liu X, Fang S, Li L, Wang Y, Jiang T. Molecular subtype impacts surgical resection in low-grade gliomas: a Chinese glioma genome Atlas database analysis. Cancer Lett. 2021;522:14–21. 34517083 10.1016/j.canlet.2021.09.008

[33] Hegi ME, Diserens A-C, Gorlia T, Hamou M-F, de Tribolet N, Weller M, Kros JM, Hainfellner JA, Mason W, Mariani L, et al. MGMT Gene silencing and benefit from Temozolomide in Glioblastoma. N Engl J Med. 2005;352(10):997–1003. 15758010 10.1056/NEJMoa043331

[34] Walsh KM, Rice T, Decker PA, Kosel ML, Kollmeyer T, Hansen HM, Zheng S, McCoy LS, Bracci PM, Anderson E, et al. Genetic variants in telomerase-related genes are associated with an older age at diagnosis in glioma patients: evidence for distinct pathways of gliomagenesis. Neuro Oncol. 2013;15(8):1041–7. 23733245 10.1093/neuonc/not051 PMC3714154

[35] Bradburn MJ, Clark TG, Love SB, Altman DG. Survival analysis part II: multivariate data analysis–an introduction to concepts and methods. Br J Cancer. 2003;89(3):431–6. 12888808 10.1038/sj.bjc.6601119 PMC2394368

[36] Hempel JM, Brendle C, Bender B, Bier G, Skardelly M, Gepfner-Tuma I, Eckert F, Ernemann U, Schittenhelm J. Contrast enhancement predicting survival in integrated molecular subtypes of diffuse glioma: an observational cohort study. J Neurooncol. 2018;139(2):373–81. 29667086 10.1007/s11060-018-2872-y

[37] Arita H, Yamasaki K, Matsushita Y, Nakamura T, Shimokawa A, Takami H, Tanaka S, Mukasa A, Shirahata M, Shimizu S, et al. A combination of TERT promoter mutation and MGMT methylation status predicts clinically relevant subgroups of newly diagnosed glioblastomas. Acta Neuropathol Commun. 2016;4(1):79. 27503138 10.1186/s40478-016-0351-2 PMC4977715

[38] Tian H, Wu H, Wu G, Xu G. Noninvasive prediction of TERT promoter mutations in High-Grade Glioma by Radiomics Analysis Based on Multiparameter MRI. Biomed Res Int. 2020;2020:1–11. 10.1155/2020/3872314 PMC7245686 32509858

[39] Ersoy TF, Keil VC, Hadizadeh DR, Gielen GH, Fimmers R, Waha A, Heidenreich B, Kumar R, Schild HH, Simon M. New prognostic factor telomerase reverse transcriptase promotor mutation presents without MR imaging biomarkers in primary glioblastoma. Neuroradiology. 2017;59(12):1223–31. 28894890 10.1007/s00234-017-1920-1

[40] Zhou H, Vallieres M, Bai HX, Su C, Tang H, Oldridge D, Zhang Z, Xiao B, Liao W, Tao Y, et al. MRI features predict survival and molecular markers in diffuse lower-grade gliomas. Neuro Oncol. 2017;19(6):862–70. 28339588 10.1093/neuonc/now256 PMC5464433

[41] Gemini L, Tortora M, Giordano P, Prudente ME, Villa A, Vargas O, Giugliano MF, Somma F, Marchello G, Chiaramonte C et al. Vasari Scoring System in Discerning between different degrees of Glioma and IDH Status Prediction: a possible machine learning application? J Imaging 2023, 9(4). 10.3390/jimaging9040075 PMC10143099 37103226

[42] Beiko J, Suki D, Hess KR, Fox BD, Cheung V, Cabral M, Shonka N, Gilbert MR, Sawaya R, Prabhu SS, et al. IDH1 mutant malignant astrocytomas are more amenable to surgical resection and have a survival benefit associated with maximal surgical resection. Neuro Oncol. 2014;16(1):81–91. 24305719 10.1093/neuonc/not159 PMC3870823

[43] Wang P, Luo C, Hong PJ, Rui WT, Wu S. The role of surgery in IDH-Wild-Type Lower-Grade Gliomas: threshold at a high extent of Resection should be pursued. Neurosurgery. 2021;88(6):1136–44. 33647953 10.1093/neuros/nyab052

[44] McGirt MJ, Chaichana KL, Gathinji M, Attenello FJ, Than K, Olivi A, Weingart JD, Brem H, Quinones-Hinojosa AR. Independent association of extent of resection with survival in patients with malignant brain astrocytoma. J Neurosurg. 2009;110(1):156–62. 18847342 10.3171/2008.4.17536

