# Supplementary material for: Recursive partitioning analysis for survival stratification and early imaging prediction of molecular biomarker in glioma patients
Source: BMC Cancer. 2024 Jul 9;24:818. doi: 10.1186/s12885-024-12542-w (PMC11232293; doi:10.1186/s12885-024-12542-w)
Supplement: Supplementary file 1 — Supplementary Material 1 [file 12885_2024_12542_MOESM1_ESM.docx]

**Supplementary**

The results for IDH and **eMethods** in this supplementary were cited from an unpublished manuscript called “Jie Z, et al. Non-enhancing margin and pial invasion in MRI can predict IDH status in glioma patients”. The citation was approved by the authors of the article.

**eMethods**

VASARI is a controlled vocabulary for characterizing the baseline visual features of human glioma images. A detailed explanation of these features can be found in the National Cancer Institute ( <https://wiki.nci.nih.gov/display/CIP/VASARI> ).

Tumor location indicates the location of lesion geographic epicenter (not all areas of involvement).

The proportion of non-contrast-enhancing tumor (nCET) represents the fraction of the entire tumor that is non-enhancing.

The proportion of enhancing tumor represents the fraction of the entire tumor that is enhancing.

The definition of non-enhancing margin, which describes the state of most of the outer non-enhancing margin of the tumor, is dichotomized into well-defined (smooth) and ill-defined (irregular).

Pial invasion indicates whether there is an enhancement of the overlying pial in continuity with enhancing or non-enhancing tumor.

Deep white matter (WM) invasion represents an extension of an enhancing or non-contrast-enhancing tumor into the internal capsule or brainstem.

Eloquent Brain: Does the geographic center or the enhancing component involve eloquent cortex (motor, language, vision) or key underlying white matter?

Thickness of enhancing margin: If most of the enhancing rim is thin, regular, and has homogenous enhancement the grade is thin. If most of the rim demonstrates nodular and/or thick enhancement, the grade is thick. If there is only solid enhancement and no rim, the grade is none.

Proportion of edema: the proportion of vasogenic edema in the entire abnormality.

**Table S1 Demographic, Clinical, Chief Complaint, Onset Symptom and VASARI MR Feature KEY of Glioma Patients with IDH Mutation and Wild Type**

| Demographic and Clinical Information | IDH-Mut^†^ (n = 66) | IDH-Wt^†^ (n = 47) | t/χ^2^ | P |
| --- | --- | --- | --- | --- |
| Age (years) | 42.00 ± 9.69, 23.00 - 66.00 | 47.64 ± 12.37, 17.00 - 68.00 | t = -2.72 | 4.0×10^-3^** |
| Gender (male/female) | 39/27 | 21/26 | χ^2^ = 2.29 | 0.13 |
| WHO Grade (II/III) | 50/16 | 21/26 | χ^2^ = 11.35 | 8.0×10^-4^** |
| Illness Duration (months) | 12.16 ± 27.13, 0.20 - 144.00 | 12.13 ± 36.12, 0.10 - 240.00 | t = 0.00 | 0.50 |
| Chief Complaint | **IDH-Mut (n = 66)** | **IDH-Wt (n = 47)** | **Z/χ^2^** | **p** |
| Sudden Disturbance or Loss of Consciousness (yes/no) | 18/48 | 10/37 | χ^2^ = 0.53 | 0.47 |
| Headache (yes/no) | 21/45 | 14/33 | χ^2^ = 0.05 | 0.82 |
| Dizziness (yes/no) | 8/58 | 7/40 | χ^2^ = 0.18 | 0.67 |
| Limb Twitching (yes/no) | 20/46 | 8/39 | χ^2^ = 2.60 | 0.11 |
| Limb Numbness (yes/no) | 6/60 | 8/39 | χ^2^ = 1.59 | 0.21 |
| Seizures (yes/no) | 7/59 | 3/44 | Z = 0.65 | 0.52 |
| Onset Symptoms | **IDH-Mut (n = 66)** | **IDH-Wt (n = 47)** | **χ^2^** | **p** |
| Epilepsy (yes/no) | 30/36 | 13/34 | χ^2^ = 3.69 | 0.05 |
| Headache (yes/no) | 20/46 | 15/32 | χ^2^ = 0.03 | 0.86 |
| Speech Impairment (yes/no) | 7/59 | 6/41 | χ^2^ = 0.13 | 0.72 |
| Visual Impairment (yes/no) | 10/56 | 14/33 | χ^2^ = 3.52 | 0.06 |
| Other Symptoms (yes/no) | 10/56 | 6/41 | χ^2^ = 0.13 | 0.72 |
| VASARI^†^ MR Feature KEY | **IDH-Mut (n = 66)** | **IDH-Wt (n = 47)** | **Z/χ^2^/OR** | **p** |
| Tumor Location (frontal/ temporal/insular/ parietal/other) | 32/9/17/8/0 | 20/10/6/7/4 | χ^2^ = 9.22 | 0.06 |
| Tumor Location  (insular/other) | 17/49 | 6/41 | χ^2^ = 2.86 | 0.09 |
| Side of Tumor Epicenter (right/center/left) | 17/2/47 | 18/0/29 | Z = 1.25 | 0.21 |
| Eloquent Brain (none/ speech motor/speech receptive/motor/vision) | 36/21/6/2/1 | 32/6/2/6/1 | χ^2^ = 9.65 | 0.05 |
| Eloquent Brain (speech motor/other) | 21/45 | 6/41 | χ^2^ = 5.48 | 0.02* |
| Enhancement Quality(none/ mild-minimal/marked-avid) | 45/8/13 | 25/8/14 | χ^2^ = 2.63 | 0.27 |
| Proportion of Contrast-Enhancing Tumor | 2.47 ± 0.75,  2.00 - 5.00 | 2.94 ± 1.21,  2.00 - 8.00 | OR = 0.59 | 0.02* |
| Proportion of nCET^†^ | 6.58 ± 1.30,  4.00 - 8.00 | 6.19 ± 1.86,  2.00 - 8.00 | OR = 1.17 | 0.20 |
| Proportion Necrosis | 2.39 ± 0.78,  2.00 - 5.00 | 2.38 ± 0.92,  2.00 - 5.00 | OR = 1.02 | 0.95 |
| Cysts (yes/no) | 20/45 | 12/35 | χ^2^ = 1.09 | 0.58 |
| T1/FLAIR Ratio | 1.45 ± 0.73,  1.00 - 3.00 | 1.51 ± 0.78,  1.00 - 3.00 | OR = 0.90 | 0.69 |
| Thickness of Enhancing Margin | 1.70 ± 1.05,  1.00 - 4.00 | 2.21 ± 1.38,  1.00 - 4.00 | OR = 0.71 | 0.03* |
| Definition of the Enhancing Margin (n.a./well-defined/ poorly-defined) | 44/8/14 | 25/11/11 | χ^2^ = 2.95 | 0.23 |
| Definition of the Non-Enhancing Margin (smooth/irregular) | 52/14 | 27/20 | χ^2^ = 5.94 | 0.01* |
| Proportion of Edema | 3.15 ± 1.22,  2.00 - 6.00 | 2.94 ± 1.39,  1.00 - 6.00 | OR = 1.14 | 0.38 |
| Edema Crosses Midline (n.a./no/yes) | 33/25/8 | 29/14/4 | χ^2^ = 1.54 | 0.46 |
| Pial Invasion (yes/no) | 30/36 | 9/38 | χ^2^ = 8.40 | 4.0×10^-3^** |
| Ependymal Invasion (yes/no) | 18/48 | 17/30 | χ^2^ = 1.02 | 0.31 |
| Deep WM^†^ Invasion (yes/no) | 20/46 | 18/29 | χ^2^ = 0.79 | 0.38 |
| nCET Tumor Crosses Midline (yes/no) | 6/58 | 5/41 | χ^2^ = 0.07 | 0.80 |
| Lesion Size | 13.09 ± 3.46,  6.00 - 18.00 | 12.79 ± 4.02,  5.00 - 18.00 | OR = 1.02 | 0.66 |

* Continuous data are shown as mean ± SD, minimum and maximum values in patients with IDH mutation and wild type with statistical significance based on two sample t test. Unordered categorical data differences in patients are represented with statistical significance based on chi-squared test (χ^2^ & p) or Fisher exact test (Z & p), while order categorical ones are calculated based on odds ratio (OR & p). *: p<0.05, **: p<0.005.

† IDH refers to isocitrate dehydrogenase, IDH-Mut refers to IDH mutation, IDH-Wt refers to IDH wild type, VASARI refers to visually accessible rembrandt images, nCET refers to non-contrast-enhancing tumor and WM refers to white matter.

**Table S2 Multivariable Logistic Regression Model for Predicting IDH Mutation**

| Variables | Odds Ratio | 95% CI | t | *P* value |
| --- | --- | --- | --- | --- |
| Age (years) | 0.96 | 0.92,1.01 | -1.74 | 0.08 |
| Gender (male/female) | 2.24 | 0.85,5.85 | 1.66 | 0.10 |
| Epilepsy (yes/no) | 2.14 | 0.66,6.93 | 1.29 | 0.20 |
| Visual Impairment (yes/no) | 0.64 | 0.19,2.09 | -0.75 | 0.45 |
| Tumor Location  (insular/other) | 2.60 | 0.61,11.00 | 1.31 | 0.19 |
| Eloquent Brain  (speech motor/other) | 2.19 | 0.57,8.46 | 1.15 | 0.25 |
| Proportion of Contrast-  Enhancing Tumor | 0.53 | 0.14,1.95 | -0.97 | 0.33 |
| Thickness of  Enhancing Margin | 0.91 | 0.35,2.32 | -0.20 | 0.84 |
| Definition of the  Non-Enhancing Margin (smooth/irregular) | 3.55 | 1.12,11.31 | 2.17 | 0.03* |
| Pial Invasion (yes/no) | 7.89 | 2.25,27.70 | 3.26 | 1.0×10^-3^** |

*: p<0.05, **: p<0.005.

**Table S3 Demographic, Clinical Factors and VASARI** **MR Feature KEY of Lower-Grade Glioma Patients with TERT Mutation and Wild Type**

| **Demographic and Clinical**  **Information** | **TERT-Wt**^†^ **(n=65)** | **TERT-Mut**^†^ **(n=43)** | **t/χ^2^/Z** | **P** |
| --- | --- | --- | --- | --- |
| **Age (years)** | 42.32±10.69  17.00-66.00 | 48.07±11.36  17.00-68.00 | t = -2.67 | 0.01* |
| **Gender (male/female)** | 33/32 | 25/18 | χ^2^ = 0.57 | 0.45 |
| **WHO**^†^ **Grade (II/III)** | 43/22 | 24/19 | χ^2^ = 1.17 | 0.28 |
| **Illness Duration (months)** | 2.00  0.10-240.00 | 2.00  0.10-144.00 | Z = 0.23 | 0.82 |
| **Chief Complaint** | **TERT-Wt (n=65)** | **TERT-Mut (n=43)** | **χ^2^/Z** | **P** |
| **Sudden Disturbance or**  **Loss of Consciousness**  **(yes/no)** | 13/52 | 14/29 | χ^2^ = 2.18 | 0.14 |
| **Headache (yes/no)** | 23/42 | 10/33 | χ^2^ = 1.79 | 0.18 |
| **Dizziness (yes/no)** | 10/55 | 5/38 | χ^2^ = 0.31 | 0.58 |
| **Limb Twitching (yes/no)** | 12/53 | 15/28 | χ^2^ = 3.72 | 0.05 |
| **Limb Numbness (yes/no)** | 5/60 | 8/35 | χ^2^ = 2.91 | 0.09 |
| **Seizures (yes/no)** | 6/59 | 4/39 | χ^2^ = 0.00 | 1.00 |
| **Speech Vague (yes/no)** | 6/59 | 0/43 | Z = -1.75 | 0.08 |
| **Onset Symptoms** | **TERT-Wt (n=65)** | **TERT-Mut (n=43)** | **χ^2^** | **P** |
| **Epilepsy (yes/no)** | 19/46 | 23/20 | χ^2^ = 6.41 | 0.01* |
| **Headache (yes/no)** | 23/42 | 10/33 | χ^2^ = 1.79 | 0.18 |
| **Speech Impairment**  **(yes/no)** | 9/56 | 4/39 | χ^2^ = 0.50 | 0.48 |
| **Visual Impairment**  **(yes/no)** | 9/56 | 13/30 | χ^2^ = 4.28 | 0.04* |
| **VASARI† MR Feature KEY** | **TERT-Wt (n=65)** | **TERT-Mut (n=43)** | **χ^2^/Z** | **P** |
| **Tumor Location**  **(frontal/temporal/insular**  **/other）** | 37/8/11/9 | 16/10/10/7 | χ^2^ = 4.55 | 0.21 |
| **Tumor Location**  **(frontal/other)** | 28/37 | 27/16 | χ^2^ = 4.02 | 0.04* |
| **Side of Tumor Epicenter**  **(right/center/left)** | 15/1/49 | 19/0/24 | Z = -2.17 | 0.03* |
| **Eloquent Brain (none/**  **speech motor/speech**  **receptive/motor/vision)** | 34/21/4/4/2 | 31/6/3/3/0 | Z = -1.44 | 0.15 |
| **Eloquent Brain**  **(speech motor/other)** | 21/44 | 6/37 | χ^2^ = 4.65 | 0.03* |
| **Enhancement Quality**  **(none/mild-minimal/ marked-avid)** | 44/9/12 | 24/7/12 | χ^2^ = 1.72 | 0.42 |
| **Proportion Enhancing**  **(<5%/6-33%/34%-67%/>67%)** | 52/12/1/0/ | 33/8/1/1 | Z = -0.32 | 0.75 |
| **Proportion nCET**^†^  **(<5%/6-33%/34-67%/68%-95%/>95%/100%)** | 5/3/8/14/4/31 | 3/1/5/9/6/19 | Z = -0.19 | 0.85 |
| **Proportion Necrosis**  **(0%/<5%/6-33%/34-67%)** | 57/0/4/4 | 34/2/5/2 | Z = -1.20 | 0.23 |
| **Cyst (no/yes)** | 43/22 | 34/9 | χ^2^ = 2.11 | 0.15 |
| **T1/Flair Ratio**  **(expansive/mixed/infiltrative)** | 42/14/9 | 32/6/5 | χ^2^ = 1.27 | 0.53 |
| **Thickness of Enhancing**  **Margin (n.a./none/thin/thick)** | 43/4/5/13 | 21/7/6/9 | Z = -1.31 | 0.19 |
| **Definition of the Enhancing**  **Margin (n.a./well-defined/**  **poorly-defined)** | 45/9/11 | 24/9/10 | χ^2^ = 2.04 | 0.36 |
| **Definition of the Non-**  **Enhancing Margin (smooth/**  **irregular)** | 51/14 | 24/19 | χ^2^ = 6.26 | 0.01* |
| **Proportion of Edema**  **(n.a./<5%/6-33%/34-67%/68-95%)** | 1/35/19/6/4 | 0/31/6/6/0 | Z = -1.75 | 0.08 |
| **Edema Crosses Midline**  **(n.a./no/yes)** | 34/24/7 | 29/11/3 | χ^2^ = 2.45 | 0.29 |
| **Pial Invasion (no/yes)** | 49/16 | 29/14 | χ^2^ = 0.81 | 0.37 |
| **Ependymal Invasion**  **(no/yes)** | 43/22 | 32/11 | χ^2^ =0.83 | 0.36 |
| **Deep WM Invasion (no/yes)** | 45/20 | 28/15 | χ^2^ = 0.20 | 0.65 |
| **nCET tumor Crosses Midline**  **(n.a./no/yes)** | 1/58/6 | 2/36/5 | Z = -0.47 | 0.64 |
| **Enhancing tumor Crosses**  **Midline (n.a./no/yes)** | 42/21/2 | 20/20/3 | Z = -1.44 | 0.15 |

Continuous data are shown as mean ± standard deviation, minimum and maximum values. The two-sample t test (t & p) or Wilcoxon test (Z & p) was used to compare continuous characteristics between TERT mutation and wild group. Statistical differences in categorical variables were represented by chi-squared test (χ^2^ & p) or Fisher exact test (Z & p). *: p<0.05, **: p<0.005.

† TERT refers to telomerase reverse transcriptase, TERT-Mut refers to telomerase reverse transcriptase mutation, TERT-Wt refers to telomerase reverse transcriptase wild type, VASARI refers to visually accessible rembrandt images, nCET refers to non-contrast-enhancing tumor and WM refers to white matter.

**Table S4 Multivariable Logistic Regression Model for Predicting TERT Mutation**

| Variables | Odds Ratio | 95% CI | Wald χ^2^ | *P* value |
| --- | --- | --- | --- | --- |
| Age (years) | 1.06 | 1.01,1.11 | 6.38 | 0.01* |
| Gender (female/male) | 0.72 | 0.27,1.89 | 0.45 | 0.50 |
| Limb Twitching (yes/no) | 0.62 | 0.12,3.13 | 0.34 | 0.56 |
| Limb Numbness (yes/no) | 0.81 | 0.10,6.56 | 0.04 | 0.84 |
| Speech Vague (yes/no) | <0.01 | <0.01,>999.99 | <0.01 | 0.92 |
| Epilepsy (yes/no) | 5.73 | 1.16,28.41 | 4.57 | 0.03* |
| Visual Impairment (yes/no) | 3.89 | 0.71,21.45 | 2.44 | 0.11 |
| Tumor Location  (frontal/other) | 0.61 | 0.23,1.63 | 0.96 | 0.32 |
| Side of Tumor Epicenter  (right/left) | 1.52 | 0.50,4.60 | <0.01 | 0.97 |
| (center/left) | <0.01 | <0.01,>999.99 | <0.01 | 0.98 |
| Eloquent Brain  (speech motor/other) | 0.54 | 0.16,1.85 | 0.97 | 0.32 |
| Definition of the  Non-Enhancing Margin (irregular/smooth) | 2.20 | 0.73,6.62 | 1.95 | 0.16 |
| Proportion of Edema | 0.74 | 0.50,1.11 | 2.12 | 0.15 |

*: p<0.05, **: p<0.005.


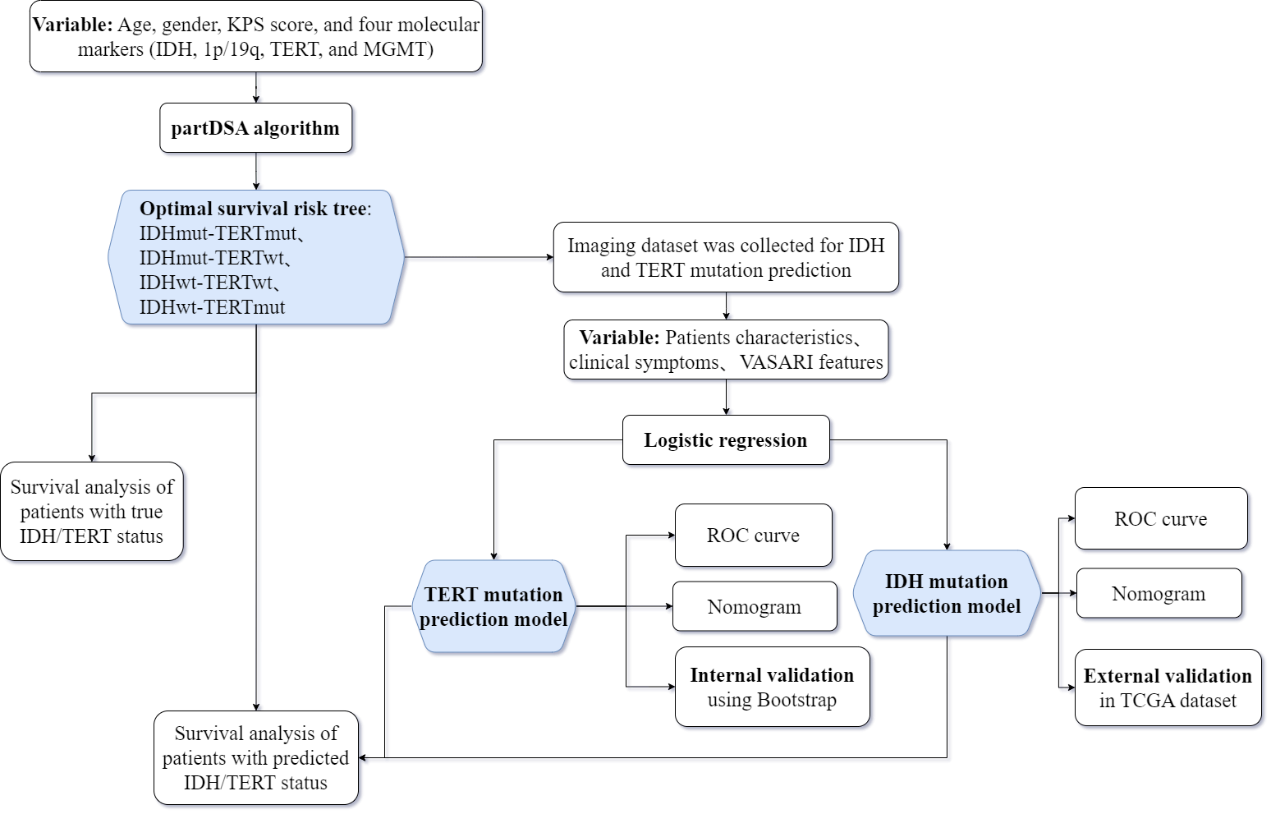


**Fig. S1 Technical roadmap**


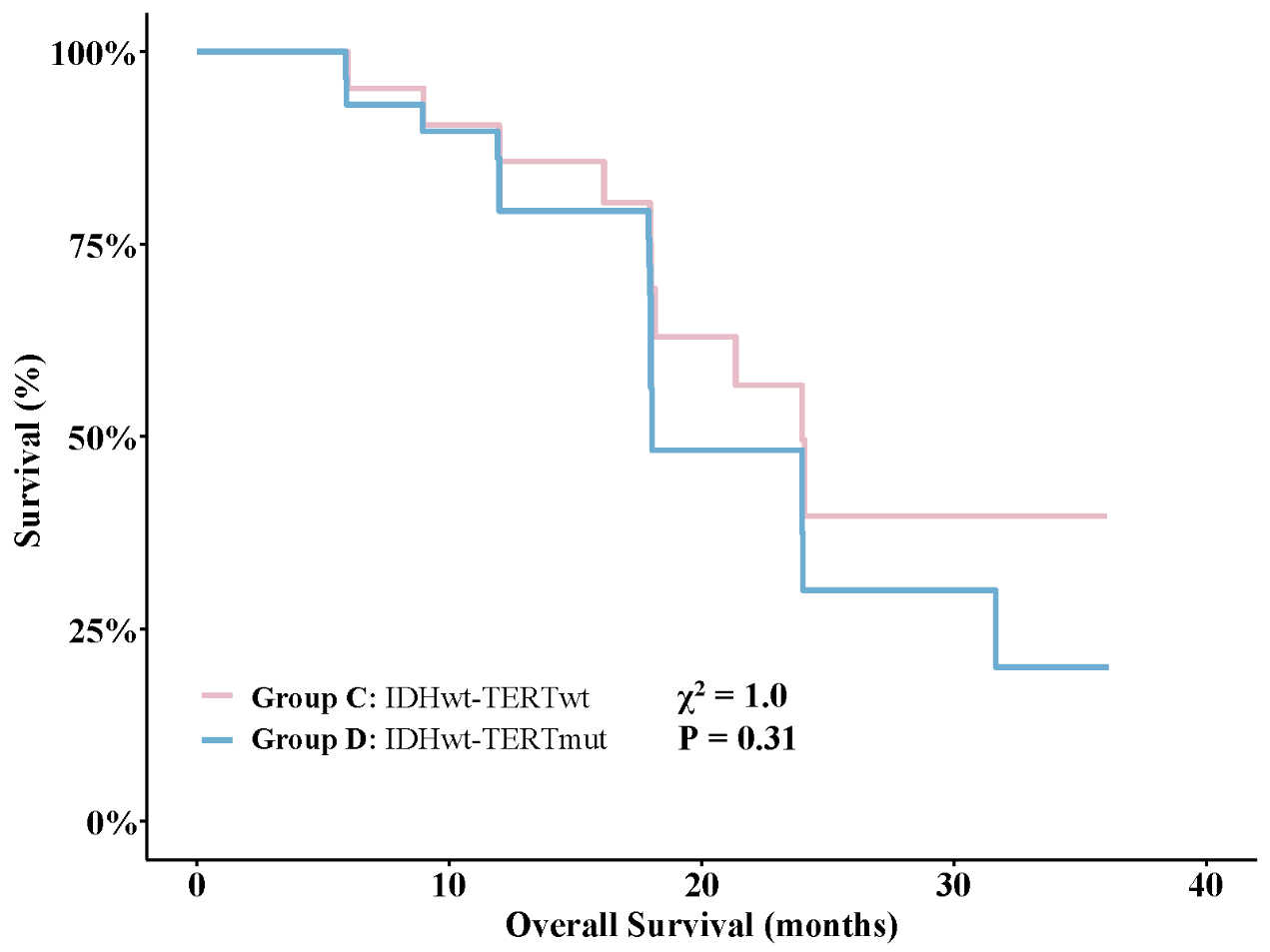


**Fig. S2 Kaplan-Meier Survival Analysis of Glioblastoma Risk Groups**

Kaplan-Meier survival curve compares the overall survival rates of two distinct risk groups within the glioblastoma cohort over a period of 40 months. Group C represents patients with wildtype IDH (IDHwt) and wildtype TERT (TERTwt), while Group D comprises patients with IDHwt and mutant TERT (TERTmut). The log-rank test shows no statistically significant difference in survival outcomes between the two groups (χ² = 1.0, P = 0.31).


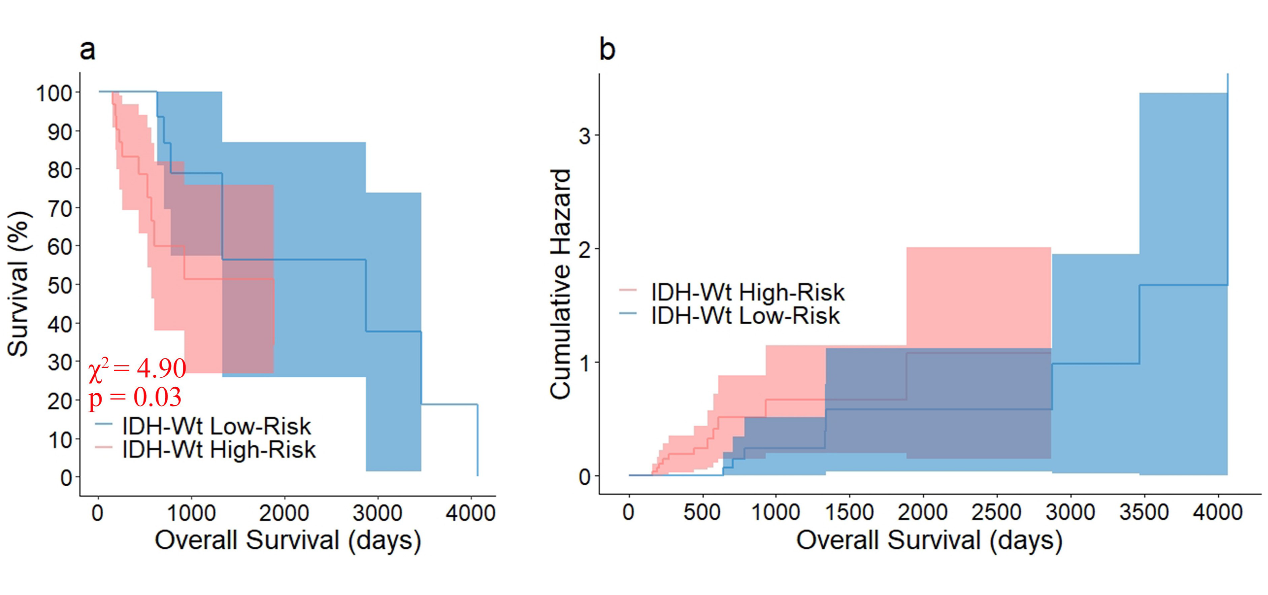


**Fig. S3 Survival Analysis and Cumulative Hazard Plot for IDH Wildtype High and Low Risk Groups of Validation Set**

(a) Kaplan-Meier survival estimate of patients with IDH wildtype high (red) and low (blue) risk groups: overall survival of IDH wildtype high-risk group was significantly shorter than that of IDH wildtype low-risk group (Log-rank test, χ2 =4.90, p=0.030). (b) Baseline cumulative hazard curve was plotted for IDH wildtype high (red) and low (blue) risk groups relevant to overall survival. Baseline cumulative hazard of IDH wildtype high-risk group was higher than that of IDH wildtype low-risk group.


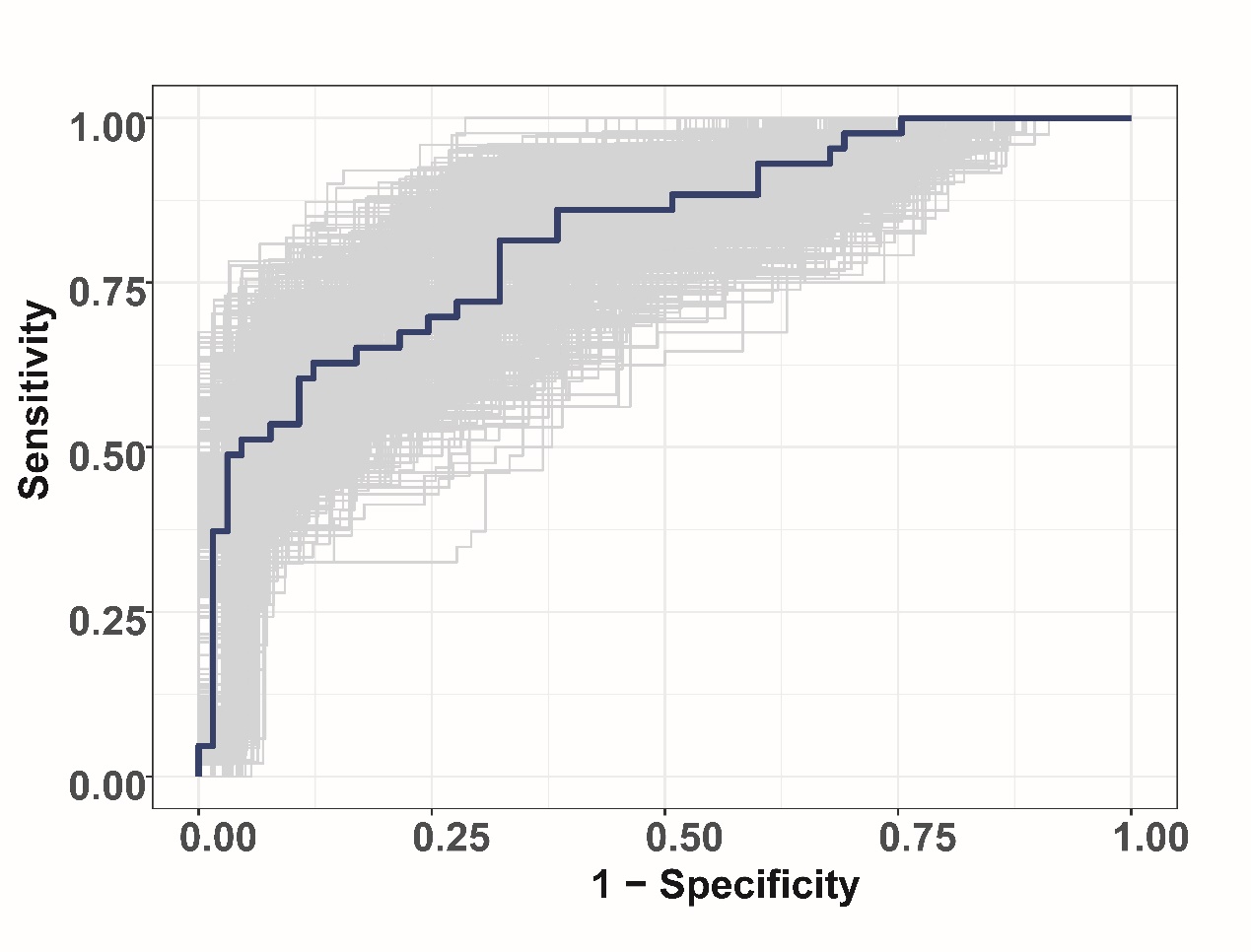


**Fig. S4 ROC curve for internal validation of TERT mutation prediction model**

The ROC curve of the TERT prediction model for the original 108 subjects is shown in blue. The samples were resampled 1000 times by Bootstrap method, and the ROC curve (grey) was plotted for each resampled sample using the prediction model, and the average AUC of resampling was still 0.82.
